# High-throughput single-сell sequencing in cancer research

**DOI:** 10.1038/s41392-022-00990-4

**Published:** 2022-05-03

**Authors:** Qingzhu Jia, Han Chu, Zheng Jin, Haixia Long, Bo Zhu

**Affiliations:** 1 Institute of Cancer, Xinqiao Hospital, Army Medical University, Chongqing, 400037 China; 2 Chongqing Key Laboratory of Immunotherapy, Chongqing, 400037 China; 3 Center of Growth, Metabolism and Aging, Key Laboratory of Bio-Resources and Eco-Environment, College of Life Sciences, Sichuan University, Chengdu, 610064 China; 4 Research Institute, GloriousMed Clinical Laboratory Co., Ltd, Shanghai, 201318 China

**Keywords:** Cancer microenvironment, Cancer

## Abstract

With advances in sequencing and instrument technology, bioinformatics analysis is being applied to batches of massive cells at single-cell resolution. High-throughput single-cell sequencing can be utilized for multi-omics characterization of tumor cells, stromal cells or infiltrated immune cells to evaluate tumor progression, responses to environmental perturbations, heterogeneous composition of the tumor microenvironment, and complex intercellular interactions between these factors. Particularly, single-cell sequencing of T cell receptors, alone or in combination with single-cell RNA sequencing, is useful in the fields of tumor immunology and immunotherapy. Clinical insights obtained from single-cell analysis are critically important for exploring the biomarkers of disease progression or antitumor treatment, as well as for guiding precise clinical decision-making for patients with malignant tumors. In this review, we summarize the clinical applications of single-cell sequencing in the fields of tumor cell evolution, tumor immunology, and tumor immunotherapy. Additionally, we analyze the tumor cell response to antitumor treatment, heterogeneity of the tumor microenvironment, and response or resistance to immune checkpoint immunotherapy. The limitations of single-cell analysis in cancer research are also discussed.

## Overview

The tumor microenvironment (TME) is a multi-component system composed of tumor cells, stromal cells, and infiltrated immune cells. Accordingly, the high-level complexity of the TME is accompanied by substantial heterogeneity at the intratumoral,^1–3^ inter-tumoral,^4^ and inter-individual^5^ levels. Within such a heterogeneous system, many pro- and antitumor cellular components or signals can regulate tumor progression and affect the efficacy of the antitumor immune response. Bulk-based omic analysis technologies provide insights into the functional mechanisms in the TME. However, owing to this extensive heterogeneity, the bulk sequencing data generated from large numbers of cell lysates provide only a “global” view of TME while obscuring the presence of cells with low abundance and highly specialized functions and ignoring universal intercellular communications.

High-throughput single-cell sequencing approaches refer to characterize a single cell at multiple levels, including their genomic,^6^ transcriptomic,^7^ epigenetic,^8^ and protein products.^9^ Comparing with traditional bulk sequencing strategies, the significant advantage of sequencing cell sequencing is evaluating heterogeneity among a population of cells, distinguishing cells with a small number and highly specified phenotype, and inferring cell behavior. In the early era of single-cell sequencing, its application is highly limited by unsatisfactory throughput and high detection cost. Nowadays, with the rapid progression in single-cell sequencing technologies, it has been widely applied in the research of various fields, particularly suitable for cancer research. Besides, emerging novel sequencing strategies continues to evolve toward a higher throughput and lower detection cost, such as single-cell combinatorial marker sequencing technique (SCI-seq),^10^ Topographic Single Cell Sequencing,^11^ or Split Pool Ligation-based Tranome sequencing.^12^ In addition, multi-omics sequencing technologies, rather than technologies that focus on single omics, provide multiple features such as DNA, RNA, protein profile for the same individual cell. Single-cell multiple sequencing technique (scCOOL-seq)^13^ enables the simultaneous examination of copy number variation, ploidy, chromatin, and DNA methylation, providing a broader view of for different cell populations. All these advanced single-cell sequencing technologies allow a broad application in the field of tumor biological and antitumor immunotherapy.

From basic/translational research to clinical practice, single-cell sequencing technology has been widely used to dissect TME composition,^14,15^ and is particularly promising in the fields of tumor immunology and immunotherapy. Resolution at the single-cell level enables identification of the immune cell population and signaling pathways that are actively involved in modulating tumor immune escape or elimination.^16^ The success of immune checkpoint blockade (ICB) immunotherapy exhibits potential for the treatment of solid tumors, enabling many patients to achieve long-term survival benefits; however, most patients do not respond well to ICB treatment.^17^ Understanding the characteristics of the baseline TME and dynamic TME changes during ICB treatment can help clarify the cellular and molecular mechanisms of ICB-driven tumor control and may reveal novel therapeutic targets for overcoming ICB resistance. By performing single-cell RNA sequencing (scRNA-Seq) alone or in combination with multi-omic strategies, the high-dimensional feature matrix at single-cell resolution can be used to infer immune cell identity and functional specification. Annotation of the cell identity merely based on scRNA-Seq may reveal a different result to the gating strategy via flow cytometry analysis. This shortage can be largely compensated by integrating cytometry and proteo-genomic data.^18^ The high-dimensional properties of scRNA-seq data allow for more refined annotation of cell subpopulations while investigating the activity of signaling pathways and inferring cellular state trajectories. Advanced bioinformatics tools have also been used to reconstruct cellular differentiation potential, determine the events driving distinct cellular states or transitions^19,20^ and construct a network of intercellular communication by exploring ligand-receptor interaction.^21,22^ The application of single-cell sequencing technology can be used for precision medicine in the clinic and to improve the outcomes of patients administered with ICB immunotherapies, such as identifying TCF7^+^CD8^+^ T cells as a predictor of positive outcomes to anti-PD-1 treatment.^23^

In this review, we summarize critical progress in single-cell sequencing analysis regarding both tumor cell behavior and tumor immunology, providing insights into tumor cell evolution and heterogeneity of the tumor immune microenvironment. The profiling of single tumor cells and immune cells has great potential to reveal novel mechanisms of resistance, immune tolerance, and relapse in individual patients with cancer, thereby it can be applied to facilitate the development of personalized and effective antitumor treatments.

### Principles of single-cell sequencing technologies, analysis pipeline, and data interpretation

**Fig. 1:**
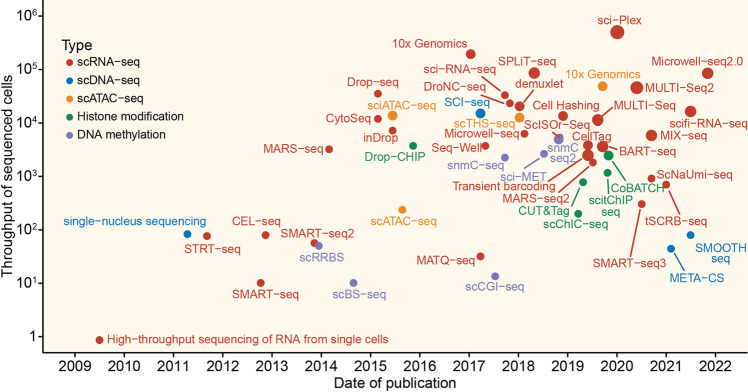
Timeline and throughput of single-cell sequencing milestones. Timeline of single-cell sequencing milestones. Scatterplot depicts the published date and throughput of sequencing for each technology. Color indicates different sequencing specifications

Since Tang et al. successfully developed high-throughput transcriptomic sequencing in 2009,^7^ many scRNA-seq protocols have been developed in the past decades (Fig. 1). In general, all protocols can be classified into two categories: full-length transcript sequencing approach and 3′/5′-end transcripts sequencing approach. The first approach attempts to produce uniform coverage of each transcript (e.g., Smart-seq2^24^) and therefore has higher sensitivity than the second, which combines unique molecular identifiers (UMIs) with transcripts to reduce technical bias during library construction (e.g., 10× Genomics^25^). Moreover, all protocols can be classified into plate- or droplet-based approaches, depending on the strategy of cell capture. The droplet-based approach is preferable for capturing large amounts of cells with low sequencing depth.^26^ However, more technical noise would be present when this strategy is used. Normally, the following steps would be involved: (a) isolate single cells from sample (blood, tissue, etc.); (b) obtain mRNA from single-cell lysis; (c) convert poly(T)-primed mRNA into cDNA with RT; (d) cDNA amplification; (e) library construction; and (f) sequencing.^27–29^ Recently, in 2017, to reduce the inefficient sample processing and technical batch effect in downstream analysis among multiple samples, a multiplexed cell capturing method for scRNA-seq was successfully developed.^30^ In brief, there are four strategies: (i) oligo-dA-based barcoding; (ii) combination of mRNA and DNA barcodes; (iii) multiplexing by viral integration; (iv) natural genetic variation.^31^

Sequencing will produce raw data in FASTQ format. The first step is gene count matrix generation, including quality control, read alignment, mapping, and gene count quantification. Cell Ranger pipeline has been developed by 10x Genomics to automatically complete the above steps for Chromium single-cell data. After obtaining the gene count matrix, the next step is processing, which includes quality control, normalization, feature selection, and dimension reduction.^32^ The goal of quality control in the processing step is to remove low-quality cells (e.g., empty cells and doublet cells). Normalization is used to remove technical bias due to the different cDNA capture efficiency and PCR amplification. In general, UMI counts are transformed to counts per million or transcripts per million.^29^ Even after removing zero count gene, the feature space for a human sample can include over 15,000 dimensions. To determine the most informative genes from the data, highly variable genes should be defined and selected. After feature selection, dimension reduction can further reduce computational burden and capture comprehensible information from complex data structure. PCA, tSNE, and UMAP are normally used in this step. However, tSNE and UMAP are not only used for dimension reduction but are also the main methods of data visualization. Downstream analysis, including clustering, annotation, trajectory analysis, and cell–cell interaction, can be performed based on well-processing data; therefore, the processing step is the most important step of scRNA-seq analysis. The clustering step involves finding and grouping cells into various populations based on similar expression patterns. Each population can be annotated as a cell type using marker-based or reference-based methods such as singleR.^33^ After annotation, differential gene expression analysis can be completed at cell-type level to determine detailed differences between cell types. In addition to cell type annotation, cell differentiation trajectory can also be inferred based on variable genes (e.g., monocle series tools^34,35^ and slingshot^36^) or RNA velocity (e.g., velocyto^20^ and scVelo^37^). In contrast to variable genes strategy, RNA velocity describes the direction and speed automatically without manually setting a root. Cell–cell communication is a common method of analysis to explore the tumor microenvironment. Two widely used platforms, Seurat^38^ and Scanpy,^39^ have been developed and integrate various computational methods that can complete most analysis steps. Meanwhile, an increasing number of algorithms are being tested to develop application for scRNA-seq data analysis.

Except transcriptome information, single-cell genome sequencing also provides new perspectives to our understanding of tumors, such as SNS,^40^ SCI-seq,^10^ SMOOTH-seq.^41^ In general, four steps have been implemented to acquire single-cell genomic sequencing data, including cell isolation, whole-genome amplification (WGA), interrogation of WGA products, and error correction.^42^ Due to only two copies of genomic DNA in human cells, WGA is one of the challenges for single-cell genome sequencing. To uniformly amplify genomic DNA in each cell, three kinds of WGA methods have been developed, including DOP-PCR (PCR-based),^43^ MDA (isothermal amplification),^44^ or MALBAC.^45^ For genomic data, variant calling is one of the most important step in the downstream analysis. Bioinformatics tools, such as SCcaller,^46^ LiRA,^47^ and Conbase^48^ have been developed for SNV detection, in addition, SCNV,^49^ HMMcopy,^50^ and Ginkgo^51^ are tools for CNV calling.

In recent years, single-cell sequencing technologies for epigenomics such as chromatin accessibility, DNA methylation, histone modification have become a possibility as well. Libraries of scATAC-seq are created from single cells that have been exposed to the Tn5 transposase by one of the following protocols: split-and-pool approach,^52^ Chromium droplet-based approach,^53^ and C1 approach.^54^ The typical pipeline includes QC, alignment, peak calling and downstream analysis, such as peak differential analysis, motif enrichment analysis, and footprinting analysis.^55^ ArchR,^56^ SnapATAC,^57^ and Signac,^58^ which consist of several algorithms, can be used in scATAC-seq data processing and analysis. Another chromatin status investigation technology is single-cell chip-seq. Drop-ChIP combines microfluidics and droplet-based sequencing protocol to obtain chromatin data.^59^ CUT&Tag uses an enzyme tethering strategy that bounds chromatin protein by appointed antibody and then generates a fusion protein as protein-Tn5 transposase.^60^ The experimental procedure of scitChIP-seq is similar to traditional ChIP-seq method with tagmentation-based library preparation strategy before canonical ChIP experiment.^61^ DROMPAplus is a ChIP-seq analysis tools with various algorithms for any species, including QC, normalization, statistical analysis, and visualization.^62^ Moreover, a few scATAC-seq tools can be used for scChIP-seq as well, such as Signac for CUT&Tag. DNA methylation is also an important aspect of epigenomics that provides information about gene expression regulation, development, and disease. The single-cell DNA methylation sequencing can be simply classified into two categories: bisulfite-based and bisulfite-free.^63^ One disadvantage of the previous one is that it cannot distinguish 5mC and 5hmC.^64^ The downstream analysis usually contains methylation calling, visualization, clustering, and methylation segmentation and differentially methylated region detection.^65^ Several mature statistical algorithms and bioinformatic tools, such as k-means, Epiclonal,^66^ NMF,^67^ and BSmooth^68^ have been applied to data analysis.

### Analysis of heterogeneity and response of tumor cells to treatment

**Fig. 2:**
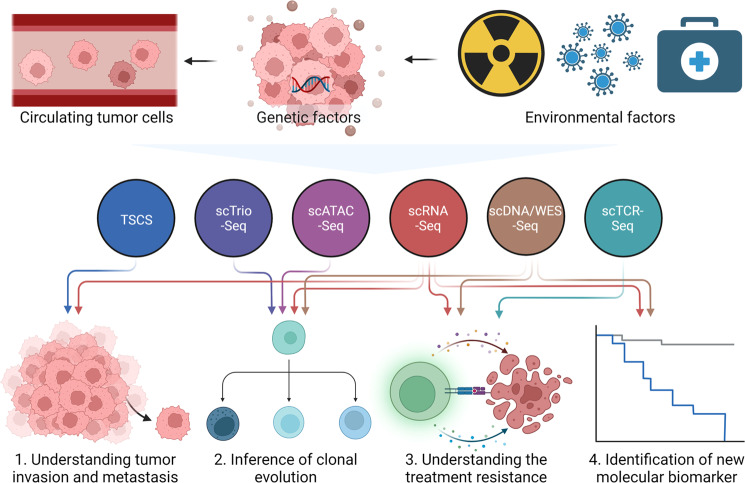
Application of single-cell omics in research of tumor cells. Tumor cells are composed of cells with various genomic alterations that influence disease progression and response to environmental perturbations and drug treatment. The characterization of high-dimensional profiling at a single tumor cell resolution facilitates the understanding of complex tumor cell behavior, clonal evolution during tumor progression, and identification of novel biomarkers for clinical application. Colored circle with arrows represents sing cell sequencing technologies and their applications in research of tumor cells

The heterogeneity of cancer at the intertumoral and intratumoral levels is consistently among the main obstacles to cancer treatment. A combination of multi-region sampling and bulk sequencing is typically used to study intratumor heterogeneity at the genome level. This method can partly reveal tumor heterogeneity but is insufficient for fully understanding lineage and temporal heterogeneity. Consequently, significant progress has been made in single-cell sequencing. After reconstructing the clonal lineage, the primary clone or subclones in the tumor lineage can be identified using single-cell technology.^69–71^ Secondly, we used tumor cell phenotypes and their signaling pathways to determine heterogeneity of epithelial–mesenchymal transition (EMT), proliferation, migration, and apoptosis. We also examined spatial heterogeneity of cancer cell clone composition at different spatial sites. During cancer occurrence and development, tumor cells evolve into different clonal lineages in response to selection pressure. Herein, we review the latest advances in the use of single-cell technology to understand the heterogeneity and evolution of tumor cells in different dimensions (Fig. 2 and Table 1).

**Table 1:** Representative studies of high throughput single-cell sequencing in research of tumor cells

Tumor	Methods	Cases	Samples	Therapy/treatment	Main findings
AML	scRNA-seq	21	Tumor/normal	-	In primitive AML cells, stemness and myeloid-related genes are co-expressed, which is related to the prognosis of patients.
	scRNA-seq	5	Tumor	-	Apoptosis and chemokine signaling are characteristics of relapsed AML, and co-targeting BCL2 and CXCR4 signaling may benefit patients.
	scATAC-seq	21	Tumor/normal	-	Chromatin accessibility can reveal the regulatory evolution in AML cells, and HOX is a key regulatory factor in the preleukemia phase.
	scDNA-seq	9	Tumor	Ivosidenib monotherapy	Ivosidenib resistance in AML patients may be caused by 2-HG restoration.
Liver cancer	scRNA-seq	2	Tumor	-	Proposes a method to quantify tumor ITH, revealing the interconnection between different components in the evolution of HCC.
	scRNA-seq	1	Tumor	-	The existence of CD24^+^ CD44^+^ subgroups suggests that there may be stemness-related HCC subclones.
	scRNA-seq	6	Tumor	-	The high expression of MLXIPL in HCC promotes the proliferation of cancer cells and inhibits their apoptosis, which is associated with poor prognosis.
	scDNA-seq	3	Tumor	-	The copy number of cancer cells only changes significantly in the early stages of cancer and ZNF717 may be the driver gene of HCC.
	scRNA-seq	19	Tumor	-	The intratumoral heterogeneity of the transcriptome of liver cancer is negatively correlated with the cytolytic activity of T cells and the prognosis of patients.
Breast cancer	scRNA-seq	6	Tumor	-	Gene regulatory networks (GRNs) at the single-cell level are of great benefit to the discovery of key regulatory factors and GRNs identified TETV6 as a key gene in TNBC.
	scRNA-seq	Patient-derived xenograft model	Patient-derived xenograft model	-	Oxidative phosphorylation is a key pathway for breast cancer metastasis, and inhibition of this pathway can significantly reduce the occurrence of metastasis.
	TSCS	10	Tumor	-	In the process of breast cancer cell invasion, the genome is relatively stable and invasive cancer is established by one or several escaped clones.
	scDNA-seq	18	Tumor/normal	-	Reveal the role of copy number alterations (CNAs) heterogeneity of breast cancer in therapeutic resistance and cancer recurrence.
	scTCR-Seq&scRNA-seq	40	Tumor	ICB	Reveal the heterogeneity of breast cancer anti-PD1 treatment response (PD1^+^ T cells, depleted T cells, and cytotoxic T cells will be cloned and expanded).
Lung cancer	scRNA-seq	8	Tumor	platinum	In small cell lung cancer, the emergence of treatment resistance is always accompanied by increased intratumoral heterogeneity.
	scRNA-seq	9	Tumor	-	The heterogeneity of lung cancer genome and transcriptome determine the heterogeneity of tumor-related pathways (including proliferation and inflammation-related), and also determines the heterogeneity of pathological characteristics.
	scRNA-seq	Kp1 cell line	Cell line	-	Small cell lung cancer has strong intratumoral heterogeneity, and this heterogeneity increases significantly after metastasis.
	scRNA-seq	7	Tumor	-	Early-stage lung adenocarcinoma has strong intratumoral heterogeneity, which leads to extremely complex interactions between different types of cells in the tumor microenvironment.
	scRNA-seq	44	Tumor	-	In the process of lung cancer metastasis, the subclones of metastatic cancer cells dominate, accompanied by the exhaustion of T cells.
	scRNA-seq	30	Tumor	TKI	TKI treatment can induce the evolution of cancer cells, and the tumors of patients with different clinical responses show different characteristics.
Colorectal cancer	scWES-seq	2	Tumor	-	Colorectal cancer is of monoclonal origin and the tumor produces new driver mutations and evolves into different subclones in the process of cancer progression.
	scWES-seq & scRNA-seq	Mouse model	Mouse model	-	The intratumoral heterogeneity of the genome of advanced colorectal cancer is reduced, and the intratumoral heterogeneity of the transcriptome is increased to adapt to changes in the environment.
	scTrio-seq	10	Tumor	-	There is strong transcriptome heterogeneity between different subclones of tumors, and DNA demethylation patterns vary greatly.
RCC	scRNA-seq	3	Tumor	-	In renal clear cell carcinoma, T cell depletion is one of the main causes of immunosuppression, which is significantly related to poor prognosis.
	scRNA-seq	8	Tumor	ICB	After ICB treatment, the immune effector cells of the responders will simultaneously up-regulate the expression of effector molecules and immunosuppressive markers.
Gastric cancer	scRNA-seq & scDNA-seq	Cell lines	Cell lines	-	The results of single-cell genome and transcriptome sequencing confirmed the strong heterogeneity within tumor cell lines.
Uveal melanomas	scRNA-seq	6	Tumor	-	HES6 plays a key role in the proliferation and metastasis of uveal melanoma and can be used as a potential therapeutic target.

- untreated

### Acute myeloid leukemia

Acute myeloid leukemias (AML) are complex ecosystems characterized by multilevel heterogeneity.^72–74^ Single-cell sequencing has revealed that AML cells are diverse, arising from the proliferation and accumulation of immature cells in bone marrow development. Mature blood cells (lymphocytes, erythrocytes, and megakaryocytes) are derived from normal hematopoietic stem cells. Results of single-cell RNA sequencing showed that a series of lineage-committed progenitor states promoted the progression of hematopoietic stem cell commitment.^75–77^ Galen et al. also found that a group of monocyte-like cells among AML cells express various immunomodulatory genes. In vitro experiments demonstrated that T cell activity was affected by this group of AML cells.^78^ AML cells can be further subdivided into naïve and differentiated cells. The heterogeneity of initial AML cells (LICs) is considered the origin of AML progression and drug resistance.^79–81^ Stetson et al. used the RNA expression profiles of 813 LICs to further explain RNA clonal evolution during AML progression. Additionally, LICs exhibited evident apoptosis and chemokine signal evolution in relapsed patients.^82^ Corces et al. determined the chromatin accessibility map of AML from the perspective of epigenetics, revealing a unique regulatory evolution in cancer cells with a further increased mutation burden. At different stages of development, AML cells also showed distinctive mixed regulome profiles.^83^

Wang et al. identified single-nucleotide polymorphisms in single tumor cells using single-cell DNA technology. They also found that patients with an IDH1 mutant in leukemia developed complex polyclonal resistance mechanisms after receiving ivosidenib monotherapy.^84^

### Liver cancer

Recent studies have revealed that primary liver cancer is one of the most heterogeneous cancers among all solid tumors.^85^ There are two main histological subtypes of liver cancer, hepatocellular carcinoma (HCC) and intrahepatic cholangiocarcinoma, which show different degrees of transcriptome heterogeneity.^86^ The heterogeneity of liver cancer is evident at the intertumoral, intratumoral, interlesional, or even intralesional levels.^87–89^ Losic et al. performed single-cell RNA sequencing of tumors in different regions, which revealed strong transcriptome heterogeneity among cells in different tumor regions. They also found that most transcription factors are not active or inactive in all tumor areas; however, one or several transcription factors are highly expressed in only one tumor area.^90^ Ho et al. observed similar intratumoral heterogeneity in liver cancer and identified a set of rare subclones rich in CD24^+^CD44^+^, which contain unique carcinogenic characteristics.^91^ Among the histological subtypes of HCC in liver cancer, Xiao et al. identified five HCC and two hepatocyte subclones with significant differences in gene expression; however, they also observed some common characteristics, including MLXIPL, which is an important marker in HCC cell trajectories.^92^ Duan et al. determined the copy number of a single HCC via single-cell whole-genome sequencing. These results indicate that HCC shows copy number variations in the early stages of liver cancer, with almost no new copy number variation introduced during tumor progression. In addition, they revealed that the origin of a specific HCC can be monoclonal or polyclonal and the intratumoral heterogeneity of polyclonal tumors is higher than that of monoclonal tumors.^93^ At the same time, Ma et al. used gene expression profiles to obtain the PCs of each tumor cell by principal component analysis, and then calculated the centroids of tumor cells in the eigenvector space (ie, the arithmetic mean of the PCs of all malignant cells in the tumor). The average distance from each tumor cell to the centroid was calculated, and this value was used as the ITH score of the tumor, which could predict the prognosis of the patient.^94^

### Breast cancer

Breast cancers are clinically stratified based on the expression of estrogen receptor, progesterone receptor, and human epidermal growth factor receptor (HER2). There are four subtypes that correlate with prognosis and define treatment strategies: luminal A, luminal B, HER2-enriched, and triple-negative breast cancer.^95^ At the transcriptional level, Her2^+^, luminal A, and luminal B subtypes also exist in malignant epithelial cells of breast cancer. Zhou et al. detected basal-like and normal-like cell subsets and revealed that ETV6 regulated different downstream genes in different subtypes to exert variable cancer-promoting roles.^96^ In a clinical human patient-derived xenograft model, single-cell RNA sequencing confirmed the transcriptional heterogeneity of primary tumors and micrometastases. Micrometastasis exhibits a specific transcriptome program that is conserved in the patient-derived xenograft model but with significantly upregulated oxidized phosphate metabolism.^97^ In breast cancer tissue, cancer cells originate from different cell lineages that evolve in parallel and possess different genomic mutations. During this evolution, in situ and aggressive subpopulations are produced.^98–100^ These subpopulations were comprehensively examined by determining the genome of a single cell. In a study of ductal carcinoma in situ, a direct genomic lineage between in situ and aggressive tumor subgroups was constructed. Before tumor invasion, most of the unique mutations or copy number variant cancer cell subtypes already exist in the duct.^101^ Copy number variation arises from breast cancer genome evolution and is a type of structural variation.^102^ Baslan et al. performed single-cell DNA sequencing to obtain genomic information for a single breast cancer cell and mapped the breast cancer genome, specifically copy number variation. Breast cancer cells exhibit both transcriptome and copy number heterogeneity. Additionally, copy number heterogeneity is significantly related to clinical or biological features such as polyploidy or HR-negative status.^103^

Bassez et al. used scTCR-Seq technology to confirm the presence of clonotype expansion in patients after PD-1 inhibitor treatment, indicating a continuous antitumor immune response; tumor cells from patients with clonotype expansion are enriched in pathways such as cell death, proteolysis, and immune signal transduction.^104^

### Lung cancer

According to histopathological results, lung cancer can be subdivided into non-small cell lung cancer (NSCLC) and small-cell lung cancer (SCLC).^105,106^ SCLC is a high-grade neuroendocrine lung carcinoma that was once considered a molecularly homogeneous malignancy. Transcriptional diversity exists among SCLC cells and the pathways involved (including EMT and C-MYC) are heterogeneous.^107^ Similarly, this heterogeneity exists in the genome of lung cancer cells (e.g., copy number variation).^108^ SCLC cells disseminate early. During cancer cell metastasis, there are dozens of transcriptome heterogeneities, which may be enhanced by metastasis. Schaff et al. revealed that when SCLC cells metastasize to the liver (the common metastatic part), their single-cell regulatory heterogeneity becomes more complicated.^109^ Lung adenocarcinoma, another subtype of lung cancer, also exhibits high heterogeneity among tumor cells, with the cells expressing high levels of proximal and distal epithelial progenitor markers. This cell heterogeneity is also evident in signaling pathways such as glycolysis, oxidative phosphorylation, and the cell cycle.^110^ Kim et al. performed scRNA-seq on paired normal or early-stage tumor tissues and metastatic tumor tissues to clarify the transcriptional heterogeneity between metastatic and primary tumor tissues; additionally, a subtype of cancer cell differentiated dissimilarly from normal epithelial cells and may predominate in metastases.^111^ Different tumor cell subclones exist in different parts of lung cancer. Sharma et al. revealed this heterogeneity and confirmed its presence in the center and edge of the tumor, with proliferation lower in the center of the tumor than at the edge.^108^ scRNA-seq analysis confirmed that after systemic targeted therapy, patients with lung cancer exhibiting different treatment responses possess cancer cells with varying characteristics. In residual disease, cancer cells exhibit characteristics of alveolar regenerative cells, indicating that the status of cancer cells changes during treatment. In patients with progressive disease, kynurenine, plasminogen, and gap-junction signaling pathways in cancer cells are significantly upregulated.^112^ In an scRNA-seq study of SCLC, after the development of platinum-based drug resistance, the heterogeneity of the tumor cell transcriptome increased significantly; however, there was almost no change in the genome (copy number).^107^

### Colorectal cancer

The molecular subtypes of colorectal cancer (CRC) are complex and highly heterogeneous. CRC is generally divided into four main consensus molecular subtypes (CMSs). CMS1 is characterized by hypermutation, microsatellite instability, and strong immune activation; CMS2 activates the epithelial, marked WNT, and MYC signal pathways; CMS3 exhibits epithelial and metabolic dysregulation; and CMS4 exhibits significant transforming growth factor-β activation, matrix invasion, and angiogenesis.^113^ Wu et al. performed single-cell exome sequencing to compare patients with normal or adenoma polyps in CRC. Both adenoma and CRC originate from the gradual accumulation of somatic mutations, particularly abnormalities in key genes such as LAMA1 (P3K-Akt signaling pathway) and ADCY3 (FGFR signaling pathway).^114^ By combining single-cell transcriptome and single-cell DNA technology, Ono et al. characterized the genome and transcriptome of a single cell; the results showed that at the exome level, tumor cell heterogeneity was not increased. After tumor transplantation, transcriptome heterogeneity increased significantly, resulting in a new subpopulation of cells exhibiting EMT signal activation.^115^ Bian et al. used single-cell triple omics sequencing to simultaneously detect the genome, transcriptome, and methylome of CRC tissues; epigenetic data showed that DNA methylation was consistent in the same tumor cell subclone but was very different in different genetic lineages.^116^

### Circulating tumor cells

Circulating tumor cells (CTCs) migrate from tumor tissues into blood vessels and play an important role in the formation of metastases. CTCs are one of the main targets of liquid biopsy.^117,118^ The amount of CTCs in the blood is very low (one part per million) and only a small amount of CTCs can be obtained from a typical blood draw.^119^ Obtaining large numbers of CTCs is difficult, which limits the utility of bulk sequencing. The application of single-cell sequencing in CTCs has introduced a new research perspective. By characterizing copy number variations or other mutational patterns by single-cell sequencing, tumor metastasis mechanisms may be uncovered. Gao et al. performed single-cell whole-genome sequencing of primary tumor cells, metastatic lymph nodes, and CTCs from colorectal cancer.^120^ Patterns of copy number variation within primary tumor cells vary greatly, with less variation among CTCs. CTCs had a similar pattern of copy number variation to metastatic lymph nodes and to a subpopulation of primary tumor cells. This suggests that the metastases were derived from a small fraction of primary tumor cells that can enter the circulatory system. Single-cell transcriptome sequencing of CTCs in gastric cancer revealed their transcriptomic heterogeneity and indicated that most gastric CTCs undergo epithelial-mesenchymal transition (EMT).^121^

Single-cell transcriptome sequencing of CTCs can help researchers understand how patients respond to treatment. For example, in the work of Miyamoto et al., single-cell transcriptome sequencing of 77 CTCs in prostate cancer demonstrated heterogeneity in expression levels of estrogen receptor genes. In a follow-up retrospective study, the non-canonical Wnt signaling pathway was found to be activated in CTCs of patients treated with androgen receptor inhibitors, suggesting potential treatment resistance.^122^ Furthermore, in small cell lung cancer, Stewart et al. acquired CTCs before platinum–etoposide treatment, at maximum response and following relapse. Subsequent single-cell transcriptome sequencing showed that CTCs from relapsed patients identified more unique clusters, implying increased transcriptomic heterogeneity in CTCs following patient resistance.^107^

### Other cancers

Several single-cell sequencing studies of patients with renal cell carcinoma (RCC) have achieved interesting results. Hu et al. performed single-cell sequencing of 12 RCC samples and nine para-tumor samples from three patients and revealed that the transcriptome of tumor cells was highly heterogeneous.^116^ In addition, the metabolism of cancer cells is abnormal, including hypoxia, lipid biosynthesis, and enrichment of localization pathways.^123^ Bi et al. mapped the single-cell RNA atlas of cancer and immune cells in patients with metastatic RCC before and after ICB treatment and revealed that the tumor cells could be divided into two subgroups: angiogenesis signals and the upregulation of immunosuppressive programs.^124^ In gastric cancer, Andor et al. determined the RNA expression profiles of thousands of single cells from gastric cancer tumor cell lines via scRNA-seq. The atlas revealed at least two subclones in each gastric cancer cell line, indicating strong transcriptome heterogeneity within these cells. The subclones of different cell lines exhibit differences in their enriched signaling pathways; however, all cells contain pathways related to genome mutation or evolution, similar to DNA repair mechanisms and metabolic pathways.^124^ In primary uveal melanomas, Pandiani et al. revealed intratumoral heterogeneity and identified HES6 as a driver of metastatic disease based on scRNA-seq.^125^ At the cellular level, single-cell sequencing can comprehensively describe cancer heterogeneity through molecular expression profiling, which cannot be achieved via bulk sequencing.^126^

### Analysis of the complex immune microenvironment

**Fig. 3:**
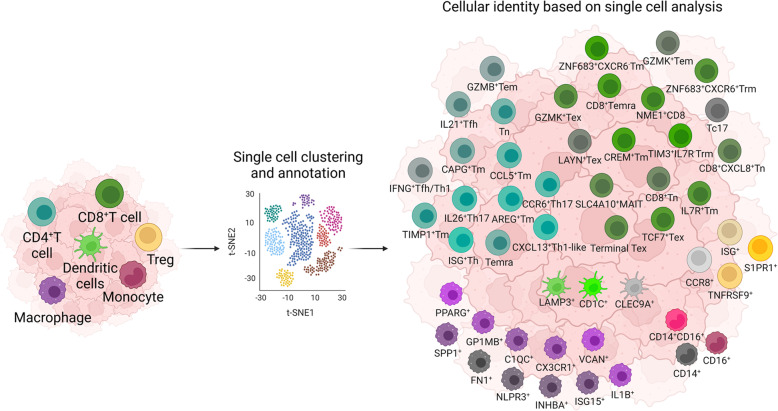
High-resolution characterization of tumor microenvironment (TME) by single-cell sequencing. Cellular architecture of the TME-infiltrated immune cells in the TME is broadly grouped using flow cytometry-based markers. Single-cell sequencing has made it possible to characterize the phenotypic heterogeneity of immune cells at the transcriptomic, proteomic, and epigenetic levels

Tumors contain not only malignant tumor cells but also various infiltrating and resident host cells, secreted factors, and extracellular proteins, collectively forming the TME.^127^ The TME is a complex and dynamic system that directly affects tumor immunity. Therefore, a comprehensive understanding of the TME is necessary for tumor therapy, particularly immunotherapy. The rapid development of single-cell omic technologies, specifically scRNA-seq, provides comprehensive information on the gene expression profile of individual cells, offering insights into the potential role of the TME. Herein, we summarize the key discoveries obtained using scRNA-seq to refine the complex cellular composition of the TME in numerous solid tumor types (Fig. 3 and Table 2).

**Table 2:** Representative studies of high throughput single-cell sequencing in research of tumor immune microenvironment

Tumor	Methods	Cases	Samples	Therapy/treatment	Main findings
NSCLC	scRNA-seq/scTCR-seq	14	Tumor/normal/blood	Treatment-naïve	Two pre-exhaustion CD8^+^ T cells were identified in NSCLC
	scRNA-seq	5	Tumor/normal	Treatment-naïve	By comparing matched normal sites, a tumor environment atlas of NSCLC was constructed
	scRNA-seq	30	Tumor/normal	Treatment-naïve	PD-1 expressing T_rm_ cells were more proliferative and cytotoxic than PD-1 expression non-T_rm_ cells in NSCLC
	scRNA-seq	44	Tumor/normal/nLN/mLN/PE/mBrain	Treatment-naïve	In addition to drawing a landscape of LUAD TME, a cancer cell cluster deviated from the normal differential trajectory and was enriched at the metastasis site
	scRNA-seq	42	Tumor/normal	Treatment-naïve	Several rare cell types in the tumor site, such as follicular dendritic cells and T helper 17 cells, were identified,
	scRNA-seq	30	Tumor/normal/mLiver/PE/mLN/mBrain	Treatment-naïve/targeted-treatment	scRNA-seq of metastatic lung cancer revealed a rich and dynamic tumor ecosystem
	scRNA-seq	7	Tumor/blood	Treatment-naïve/chemo-treated	Mapping tumor-infiltrating myeloid cells in patients with NSCLC by scRNA-seq
BC	scRNA-seq/WES/RNA-seq	11	Tumor	Treatment-naïve	Highly intratumoral heterogeneity tumor environment was shaped by tumor cells and immune cells in breast cancer
	scRNA-seq/scTCR-seq	8	Tumor/normal/blood/LN	Treatment-naïve	Supported continuous activation model of T cells and disagreed with macrophage polarization model in breast cancer
	scRNA-seq/scTCR-seq/RNA-seq	123 (2 for scRNA-seq)	Tumor	Treatment-naïve	Tissue-resident memory T cells were enriched in breast cancer and expressed high levels of immune molecule and effector proteins
	scRNA-seq/scTCR-seq	14	Tumor	Treatment-naïve	Drew an atlas of tumor microenvironment of TNBC and defined a novel TCR-expressing macrophage
HCC	scRNA-seq/scTCR-seq	6	Tumor/normal/blood	Treatment-naïve	Eleven T cell clusters were defined, and several specific clusters such as exhausted CD8^+^ T cells were enriched in the HCC tumor site
	scRNA-seq	19	Tumor	Treatment-naïve	Hypoxia-dependent VEGF was associated with tumor diversity and TME polarization. The cytotoxic capacity of T cells was lower in higher heterogeneity HCC
	scRNA-seq/scTCR-seq/CyTOF	13	Tumor/non-tumor/leading-edge	Treatment-naïve	Defined tumor-associated CD4/CD8 double-positive T cells in HCC and systematically analyzed the function of PD-1^+^ DPT in HCC
	scRNA-seq	5	Tumor	Treatment-naïve	Constructed a human liver cancer landscape in single-cell resolution
	scRNA-seq/scTCR-seq	16	Tumor/normal/blood/ascites/nLN	Treatment-naïve	Drew an immune cell atlas of HCC. A novel DC cluster with high expression of LAMP3 was defined, and it may regulate multiple immune cells
	scRNA-seq/RNA-seq	48 (6 for scRNA-seq)	Tumor/normal/margin tissue	Treatment-naïve	Comprehensively analyzed tumor ILC composition and found that patients with higher IL-33 expression exhibited a higher ILC2/ILC1 ratio, indicating better prognosis
Melanoma	scRNA-seq/WES	19	Tumor	Treatment-naïve	Demonstrated the tumor environment ecosystem and how scRNA-seq offers insights into the results
	scRNA-seq/scTCR-seq	25	Tumor	Treatment-naïve	scRNA-seq analysis revealed gradual T cell dysfunction in melanoma and exhausted CD8^+^ T cells were proliferative and expanded cell cluster
	scRNA-seq/scDNA-seq/TCR-seq	11	Tumor	Treatment-naïve	Novel CD8^+^ T cells were observed with predominantly expressing LAG-3, rather than PD-1 or CTLA-4
CRC	scRNA-seq	11	Tumor/normal	Treatment-naïve	Two distinct CAF clusters were identified. Tumor-enriched CAF clusters highly expressed EMT-related genes
	scRNA-seq/scTCR-seq	12	Tumor/normal/blood	Treatment-naïve	Developed STARTRAC indices to analyze the relationship, function, and clonality of 20 identified T cell clusters in CRC
	scRNA-seq	1	Tumor/normal	Treatment-naïve	Expression clustering identified six gene modules, and functional enrichment was associated with T cells and cancer cells
	scRNA-seq/scTCR-seq	18	Tumor/normal/blood	Treatment-naïve	Two specific macrophages and cDC clusters, which played a key role of cellular crosstalk in the CRC TME were identified
	scRNA-seq	29	Tumor/normal	Treatment-naïve	Provided the tumor environment landscape and intercellular communications in CRC
Bladder cancer	scRNA-seq/scTCR-seq	7	Tumor/normal	Treatment-naïve	Found multiple tumor-specific CD4 + T cells. Cytotoxic CD4 + T cells in tumor site were highly proliferative and could kill autologous tumors in an MHC class II-dependent manner
	scRNA-seq	11	Tumor/blood	Treatment-naïve	Constructed a cell atlas in bladder cancer and provided deep insights into the tumor microenvironment
ICC	scRNA-seq	4	Tumor/adjacent tissue	Treatment-naïve/recurrent	Demonstrated intertumor heterogeneity of human ICC and provided information on intercellular crosstalk between tumor cells and vCAFs
Gastric cancer	scRNA-seq	8	Tumor/metaplasia/normal/blood	Treatment-naïve	Comparing to normal site, scRNA-seq analysis revealed tumor-enrichment immune cells, transcriptional states, and intercellular interactions in gastric cancer
ccRCC	Mass cytometry/RNA-seq	47	Tumor/normal	Treatment-naïve/treatment	By profiling 3.5 million single cells, the study developed an in-depth tumor microenvironment atlas of ccRCC and revealed potential biomarkers for therapy strategies
Nasopharyngeal	scRNA-seq/scTCR-seq	3	Tumor	Treatment-naïve	Provided insights into the tumor microenvironment at single-cell resolution and revealed heterogeneity of immune cells and various functional T cell clusters in NPC
Ovarian cancer	scRNA-seq	9	Tumor/normal/benign	Treatment-naïve	Identified specific cell clusters enriched in different grades of ovarian cancer
Pan-cancer	scRNA-seq/scTCR-seq	14	Tumor/normal/blood	Treatment-naïve	Together with published data, demonstrated that non-exhausted T cells from outside of the tumor can replace exhaustion T cells in responsive patients
	scRNA-seq	20	Tumor/normal	Treatment-naïve	Provided an integration immune cell atlas across lung, breast, and ovarian cancers and revealed the complexity of stromal cells in different cancer types
	scRNA-seq/RNA-seq/Exome-seq	48	Tumor/normal/blood/LN	Treatment-naïve	Drew a pan-cancer myeloid landscape via scRNA-seq. Different sources of LAMP3^+^ DCs exhibited various transcription expression patterns, and TAMs were also diverse across cancer types

### NSCLC

Non-malignant cells in the NSCLC TME affect both tumor-promoting and tumor-suppressive activities.^127–130^ The identification of components, particularly the cell populations and their function in the TME, has yielded potential strategies for immunotherapy. Previous studies have revealed that treatment efficacy varies and partly depends on the number and properties of tumor-infiltrating lymphocytes.^131–133^

Several recent studies have focused on the complexity of T cells in NSCLC. Guo et al. first used a full-length single-cell mRNA-seq technique, Smart-seq2, to evaluate NSCLC biopsies from treatment-naïve patients to determine the baseline landscape of tumor-infiltrating lymphocytes.^134^ In addition to conventional CD4^+^ and CD8^+^ T cell clusters, two novel CD8^+^ T cell clusters were observed and defined as “pre-exhausted” cells, which exhibited low expression of exhaustion markers. One subset was significantly abundant in NSCLC and exhibited high expression of ZNF683, suggesting that it functions in NSCLC immunity.^134^ Another pre-exhausted subset was characterized by high expression levels of GZMK, which was associated with the “effector memory” phenotype.^135^ However, GZMK^+^CD8^+^ T cells with intermediate exhaustion markers are likely transferred into exhausted T cells.^136^ By comparing intratumoral and para-tumoral lung-derived T cells, Lambrechts et al. confirmed elevated glycolysis and suppressed oxidative phosphorylation coherent between T cell clusters.^137^ Moreover, the authors found that a CD8^+^ T cell cluster enriched at the tumor site exhibited strong IFN-γ and IFN-α responses, high granzyme expression, and expressed high levels of exhaustion markers (LAG3, TIGIT, PDCD1, and CTLA4). In another study, Clarke et al. reported a TIM3^+^IL7R-tissue-resident memory T cells (T rm) subset uniquely present at the tumor site and expressing high levels of PD-1 and other molecules linked to inhibitory functions; however, functionality analysis revealed that these cells were not exhausted.^138^ Compared with non-T_rm_ cells, PD-1-expressing T_rm_ cells were associated with the key effector cytokines IL-2, TNF, and IFN-γ as well as granzyme molecules. In addition to CD8^+^ T cells, recent studies demonstrated the depletion of natural killer cells^139^ and increased emergence of Tregs at the primary tumor site compared to at the normal site.^128,137,140^ Tregs persist in tumors and metastasis sites to suppress antitumor immunity in NSCLC.^140^ Wu et al. first reported a rare T cell cluster, Th17, in NSCLC with a high expression level of KLRB1 and observed a transitional phenotype from naïve cells to Tregs.^141^

Myeloid cells also play a pivotal role in tissue homeostasis and inflammation in lungs.^140^ Unsupervised clustering analysis and *t*-SNE plotting of myeloid lineage cells revealed a substantial difference between intra-tumoral and non-tumoral lung macrophages, indicating that they have a completely different composition.^137^ These results correspond with those of Kim et al.^140^ who revealed that tumor-associated macrophages in tumor sites are primarily derived from monocyte-macrophages rather than from tissue-resident macrophages. Moreover, the upregulation of the transcription factors IRF2, IRF7, IRF9, and STAT2 and downregulation of inflammatory enhancers such as Fos/Jun supported M2 polarization of tumor macrophages in NSCLC. Another study revealed that macrophages in treatment-naïve patients exhibit M2 polarization.^142^ Zilionis et al. mapped the myeloid landscape in human and murine lung tumors, revealing conserved populations across individuals and species;^143^ additionally, their work revealed limited overlap of myeloid populations between blood and tumors in patients. One subset of dendritic cells (plasmacytoid dendritic cells [DCs]) was rarely found in normal lung sites compared to tumor sites and metastatic lymph nodes and exhibited an immunosuppressive phenotype with the upregulation of LILR and GZMB but loss of CD86, CD83, CD80, and LAMP3.^140^ A rare cell type, defined as follicular DCs, was identified by Wu et al.^141^ Furthermore, Lambrechts et al. detected a fibroblast cluster highly expressing COL10A1 that was strongly enriched at the tumor site and exhibited a strong EMT signal.^137^

### Breast cancer

The function of tumor-infiltrating lymphocytes in breast cancer remains unclear. Some studies have suggested that increasing number of tumor-infiltrating lymphocytes can improve patient survival, whereas other studies revealed contrasting results.^144,145^ scRNA-seq provided a cell-level landscape that can be used to further investigate the function of tumor-infiltrating lymphocytes in breast cancer.

A previous study of primary breast cancer grouped 175 immune cells into three clusters: T cells, B cells, and M2 macrophages, all exhibiting an immunosuppressive phenotype.^146^ In contrast, Azizi et al. profiled 47,016 CD45^+^ cells from treatment-naïve patients with breast cancer and revealed significant heterogeneity for both lymphoid and myeloid cells.^147^ Moreover, the observed continuum of T cell states indicated that canonical classification of T cell clusters oversimplifies the tumor environment of breast cancer. Treg clusters expressed similar patterns of anti-inflammatory, exhaustion, hypoxia, and metabolism genes, suggesting that the functions of different clusters are inconsistent. Savas et al. identified a T_rm_-like cluster highly expressing CD103 and occupying a large proportion of CD8^+^ T cells.^148^ This cluster expressed granzyme and immune checkpoint molecules, suggesting their cytotoxic ability and proinflammatory potential.

Breast cancer cells secrete various cytokines that influence myeloid cell differentiation and suppress antitumor immunity.^149,150^ Azizi et al. revealed that gene expression in M1 and M2 macrophages was positively correlated and frequently expressed in the same cells.^147^ This finding indicates that the polarization model cannot be applied to tumor-associated macrophages. To better understand the characteristics and capacity of myeloid-derived suppressor cells (MDSCs), Alshetaiwi et al. established an MDSC-specific gene set between G- and M-MDSCs. This is unique to normal myeloid counterparts from a murine breast cancer model; however, these results can also be transferred to the human context, with a conserved state of MDSCs between mouse and human suggested.^151^ IL1B, ARG2, CD84, WFDC17, and chemokine receptors (e.g., CCR2 and CXCR2) were included in this gene set, indicating an immunosuppressive function. Particularly, CD84^+^ MDSCs capable of T cell suppression and increased reactive oxygen species production were observed. Moreover, the spleen was detected as a major site of MDSC emergence in breast cancer.

Qiu et al. collected 9683 tumor-infiltrating immune cells from treatment-naïve patients with TNBC and identified several novel cell clusters, such as CD8^+^CXCL8^+^ naïve T cells. CXCL8 was observed primarily during the production of naïve CD4^+^ cells in the human peripheral blood or in infants.^152^ Consistent with its function in CD4^+^ T cells,^153^ differential gene expression and pathway enrichment analyses suggest that the cluster mediates neutrophil migration and activates MAPK/extracellular signal-regulated kinase pathways, which contribute to tumor growth. Notably, the number of double-negative T cells (CD3^+^CD4^-^ CD8^-^) accounted for ~31.0% of all T cells in breast cancer but only 1–5% in healthy humans.^154,155^ Double-negative T cells play a key role in inflammation and autoimmunity.^152,153^ However, three independent clusters with high levels of effector markers (GZMA, GZMB, and IFN-γ), regulatory markers (FOXP3 and IL2RA), and naïve markers (CCR7) indicate that double-negative T cell function is important and complex in the TNBC microenvironment. Notably, a novel cluster of CD3- and T cell receptor (TCR)-positive macrophages was first observed in the TME of breast cancer. The upregulation of TCR signaling and cytotoxic effect of genes compared to TCR- macrophages indicates that these macrophages exert partial T cell functions. At the single-cell level, authors also observed a “pre-exhaustion” T cell cluster and high positive correlation of gene expression between M1 and M2 macrophages, supporting previous results.^147^

### HCC

The functions of specialized immune cells in HCC, such as Kupffer cells (macrophages), innate lymphoid cells, and various T cells, are not well-understood.^156,157^ Researchers have recently focused on investigating immune cells using scRNA-seq.

Atlas analysis in HCC classified infiltrating T cells into 11 large subsets.^158^ Most clusters have been reported in other cancers, such as “pre-exhausted” T cells in NSCLC and breast cancer.^134^ A unique CD8^+^ cluster with positive expression of FOXP3 was defined as a Treg-like population and exhibited both suppressive and cell-killing characteristics. Zheng et al. also found that LAYN plays a regulatory role not only in Tregs,^159^ but also in CD8^+^ T cells in HCC. Furthermore, Ma et al. described the different functions of highly and poorly heterogeneous (Div-high and -low, defined by the authors) HCC based on the analysis of the gene expression pattern, pathway enrichment, and T cell cluster distribution.^86^ Cytotoxic-related genes (e.g., GZMA and GZMB) and immune checkpoint molecules (IFNG, PDCD1, and NKG7) were highly expressed in Div-low compared to in Div-high HCC. The upregulated pathways in Div-high tumors included EMT and myogenesis, whereas allograft rejection, oxidative phosphorylation, and fatty acid oxidation were upregulated in Div-low tumors. A more detailed analysis revealed that immunosuppressive Tregs were considerably higher in Div-high than in Div-low HCC. By evaluating a combination of tumor, leading edge, and normal samples, Zheng et al. found that the CD4 and CD8 double-positive T (DPT) cell cluster was enriched in the leading-edge region;^160^ however, this cluster generally exists in the thymus.^161^ The DPT cluster was divided into 11 subclusters based on canonical markers including cytotoxic DPT, memory DPT, activated DPT, NK-like DPT, MAIT-like DPT, and exhaustion DPT. Trajectory analysis revealed that the DPT was functional and well-differentiated T cells and in conjunction with TCR data, showed a common ancestry of PD-1^+^DPT and PD-1^+^CD8^+^ T cells.^160^

Several specialized and unique macrophage subsets have been reported in recent studies. MacParland et al. observed two distinct CD68^+^ macrophages in primary HCC samples, one with high expression levels of inflammatory markers (LYZ, CSTA, and CD74)^162^ and another with enriched expression of immunoregulator genes (e.g., MARCO, VSIG4, and CD163).^163^ Aizarani et al. also identified new subsets of Kupffer cells expressing CD1C or LIRB5, which shared gene expression and pathways with endothelial cells, suggesting functional cooperation.^164^ Zhang et al. found two distinct tumor-associated macrophages^165^ with high expression levels of SLC40A1 and GPNMB, by combining 10x Genomics and Smart-seq2.^166^ Consistent with recent studies of iron metabolism in macrophages,^167,168^ SLC40A1 encodes the iron exporter ferroportin and promotes proinflammatory cytokines, including IL-23 and IL-6, but suppresses IL1β production. In contrast to classical DCs (cDCs), LAMP3^+^ DCs may represent a common DC cluster with several interesting characteristics.^166^ Many ligands for T and NK cell receptors were expressed, suggesting that these receptors play immunoregulatory roles in lymphocytes. Moreover, signature genes of LAMP3^+^ DCs were strongly correlated with the signature of Treg or Tex, indicating that DCs contribute to T cell dysfunction. In contrast, LAMP3^+^ DCs can migrate from HCC tumors to lymph nodes and prime T cell migration to the tumor site.^166^

Due to the low abundance of innate immune lymphocytes, the studies on innate lymphoid cells (ILCs) in human liver cancer are limited. Heinrich et al. performed scRNA-seq analysis to draw a landscape and determine the role of ILCs in human HCC.^169^ Four canonical cell types, including ILC1, ILC2, ILC3, and NK-like, were identified; the authors also defined a CD127-NK-like cluster with an intermediate status between NK cells and ILC1s in HCC. Importantly, the first study that described the details of the conversion of ILC3s to ILC2s and ILC1s to ILC2s^169^ suggested IL-4 as a cytokine that drives such a transition.^170,171^ Overall, these findings demonstrate the diversity of immunoregulatory mechanisms in the HCC TME.

### Melanoma

Recently, scRNA-Seq began to dissect TME heterogeneity in melanoma. Tirosh et al. identified core exhaustion signatures of T cells, including upregulation of coinhibitory (TIGIT) and costimulatory (TNFRSF9/4-1BB and CD27) receptors.^172^ In line with the findings for NSCLC and breast cancer,^134,147^ Li et al. noted that dysfunctional CD8^+^ T cells transitioned from early effector cells.^173^ Analysis of the TCR in peripheral blood mononuclear cells revealed dysfunctional processes at the tumor site. Additionally, dysfunctional CD8^+^ T cells are highly proliferative and dynamic. In contrast to the transitional state of dysfunctional CD8^+^ T cells, cytotoxic CD8^+^ T cells formed a discrete state with independent signatures and were unlinked with dysfunctional CD8^+^ T cells. Durante et al. drew a TME atlas at single-cell resolution in uveal melanoma and identified an unrecognized CD8^+^ T cell, which primarily expressed LAG3, an exhaustion-associated immune checkpoint molecule in CRC.^174^

By analyzing 333 individual DCs and monocytes from metastatic melanoma, Nirschl et al. revealed that homeostatic modules were enriched in monocytes and DCs and were positively correlated with IFN-γ signatures.^175^ Moreover, SOCS2, a member of the SOCS family that uniquely degrade all other members,^176,177^ was induced by IFN-γ, which is present on monocytes and part of a tissue signature during melanoma formation.

### CRC

In contrast to previously summarized cancers, immunotherapy has shown limited advances in the treatment of patients with CRC,^178^ likely due to incomplete understanding of the TME in CRC.^179,180^ scRNA-seq is a powerful technique that can improve the capacity to excavate and understand the complexity of the TME in CRC.

Similar to NSCLC^134^ and HCC,^158^ Zhang et al. profiled the T cell atlas in CRC using Smart-seq2 and TCR-seq and identified 20 unique T cell clusters, including typical CD8^+^ and CD4^+^ T cell clusters. In contrast, additional T cell clusters, including Th17, follicular T helper cells, follicular T regulatory cells, and two CD8^+^ T cell clusters were identified in CRC.^181^ Among them, Tex cells, two IFNG^+^ Th1 cells, and one Treg cluster were enriched in the tumor. Focusing on the tumor subtypes, CXCL13^+^BHLHE40^+^ Th1-like cells were enriched in microsatellite instability tumors, whereas Th17 cells primarily existed in microsatellite stable tumors. Lee et al. also found that Th17 cells and Tregs were predominantly present at the tumor site, whereas γδT cells were enriched at the normal tissue site.^182^ Zhang et al. observed that CD8^+^ T cells, Th1/Th2 cells, and memory T cells were increased at the tumor site, whereas CD4^+^ T cells and Tregs were decreased.^183^ Based on enrichment analysis results, the imbalance in T cell clusters may be affected by T cell proliferation, activation/differentiation, and TCR signaling.

Lee et al. suggested that the immunosuppressive function of myeloid cells is enhanced because of their expansion in CRC.^181^ SPP1^+^ macrophages were more abundant in tumor sites than in normal sites. Previous studies have revealed that SPP1^+^ macrophages play a central role in both the pro- and anti-inflammatory phenotype,^184–186^ which is consistent with the findings of Lee et al. In addition to T cells, Zhang et al. combined 10x genomics and Smart-seq2 to draw a transcriptome landscape of CRC immune cells, focusing on myeloid cells.^187^ Two distinct TAM populations were identified: C1QC^+^ and SPP1^+^ TAM. Consistent with previous studies of other cancers, these clusters could not be explained by either the M1 or M2 phenotype.^147,188^ C1QC^+^ TAM was primarily associated with phagocytosis and antigen presentation, whereas SPP1^+^ TAM was significantly enriched in angiogenesis regulation. Moreover, compared to the normal site, SPP1^+^ TAM exhibited greater enrichment at the tumor site. In addition to SPP1, Zhang et al. found that GPNMB, which was reported to mediate MDSCs and inhibit T cells,^189^ was highly expressed in granulocytes.^183^ The authors also revealed that IL-17 signaling and ferroptosis pathways were enriched in granulocytes at the tumor site. The IL-17 pathway may play a role in CRC liver metastasis and the ferroptosis pathway has been reported to mediate reactive oxygen species production and p53 downstream effectors during cancer cell death.^190,191^

Li et al. developed a novel algorithm named as reference component analysis and identified two distinct clusters of cancer-associated fibroblasts (CAFs), CAF-A and CAF-B.^192^ CAF-A may be an intermediate cluster between normal fibroblasts and CAF-B, as it expresses genes related to extracellular matrix remodeling, whereas CAF-B expresses known markers of activated myofibroblasts. In addition, EMT-related genes are highly expressed in CAFs.

### Other cancers

With the widespread use of scRNA-seq technology, an increasing number of TME from different cancer types have been studied. Oh et al. found that the composition of CD8^+^ T cells did not differ between bladder tumor and non-malignant tissues.^193^ In contrast, tumor-specific Tregs were identified with high expression levels of IL2RA and immune checkpoint molecules. Multiple cytotoxic CD4^+^ T cell clusters were clearly defined using canonical markers, suggesting that these cells kill autologous tumor cells. Chen et al. found that monocytes were enriched in the M2 state of the bladder tumor region; moreover, a cluster of DCs with high LAMP3 expression was correlated with Treg recruitment, indicating the regulation of immunosuppressive formation.^194^ Zhang et al. employed scRNA-seq to create the transcriptomic landscape of intrahepatic cholangiocarcinoma and identified six distinct types of fibroblasts.^195^ The main fibroblast cluster was vascular CAFs, which expressed high levels of IL-6, thereby enhancing the malignancy of the tumor cells. In gastric cancer, Sathe et al. found that two cytotoxic T lymphocyte clusters highly expressed exhaustion markers, with one cluster exhibiting higher proliferation potential than the other. Macrophages cannot simply be classified as M1 or M2 macrophages because the canonical markers are co-expressed in the same cluster. Additionally, the authors identified a cluster of DCs enriched at the tumor site expressing chemokines such as CCL22, CCL17, CCL19, and IL32, which are associated with naïve T cell recruitment.^196^ By profiling 3.5 million cells from 73 patients with clear cell RCC (ccRCC), Chevrier et al. constructed an immune atlas with 22T cell clusters and 17 macrophage clusters. Moreover, the authors found that CD38, a mediator of nitric oxide,^197^ is a potential exhaustion marker of T cells in ccRCC. As the first landscape of TME in nasopharyngeal carcinoma (NPC), Zhao et al. observed that the proportion of B cells was higher than that of T cells, particularly in Epstein–Barr virus-negative patients.^198^ A study reported that malignant cells secrete mediators (e.g., miR-21) to expand B cells, which suppress CD8^+^ T cells.^199^ Thus, B cells may be a useful immunotherapeutic target in NPC.^198^ Shih et al. recently revealed that myeloid cells of both primary and metastatic ovarian cells and fibroblasts of metastatic ovarian cells produced increased levels of secreted factors, indicating that these cells play important roles in tumor growth.^200^ Additionally, B cells and T cells did not express high levels of pro-tumor genes such as IL-10, STAT3, and CCL22 but expressed genes that influenced complement pathways.

### Pan-cancer analysis

Several studies have recently concentrated on excavating the TME with multiple tumors and integrating scRNA-seq data. Wu et al. identified two T cell clusters that did not match published studies; they collected 141,623 T cells from four different tumor types, including NSCLC, uterine corpus endometrial carcinoma, CRC, and RCC.^135^ One cluster expressed high levels of the long noncoding RNA MALAT1 and chromatin remodeling enzyme CHD1. Another cluster was enriched in mitosis genes. Neither cluster had high scores on T_rm_ cell signature, which was considered as the origin of exhausted T cells.

Qian et al. collected 233,591 single cells from lung, colon, ovarian, and breast cancer tumors.^201^ Overall, authors identified 22 tumor-specific clusters, including mast cells, germinal center-independent B cells, and neutrophils. The new cDC2 subset expressed CD1C and Langerhans cell-specific markers, such as CD207 and CD1A. In T cells, clustering was not affected by CCA, suggesting that T cell distribution was not tumor-specific. However, both B cells and T cells were more enriched in LC than in OV and CRC, likely because LC is a hot tumor.

Zhang et al. collected myeloid cells from 15 different types of tumors and identified distinct features of myeloid cells across the tumor types.^202^ However, NPC possessed a higher proportion of mast cells and was the only tumor with higher TNF^+^ mast cells than VEGFA^+^ , indicating a stronger antitumor function. The authors also confirmed a previously reported cDC subset,^166^ LAMP3^+^ cDCs, which widely exist across all 15 tumor types. Although both conventional type 1 dendritic cells (cDC1) and cDC2 could differentiate into cDC3, more LAMP3^+^ cDCs were derived from cDC1, except in pancreatic adenocarcinoma and NPC. cDC1-derived LAMP3^+^ cDCs highly expressed IL12B and BTLA, which induced the differentiation of T helper 1 cells and Treg cells, respectively.^203,204^ In contrast, CCL17, a chemokine that recruits Tregs into tumors,^205^ was specifically expressed by cDC2-derived LAMP3^+^ DCs. Macrophage clusters from different tumor types were diverse; additionally, SPP1^+^, C1QC^+^, ISG15^+^, and FN1^+^ TAMs were primarily enriched at the tumor site. The authors also found that both M1 and M2 gene signatures were co-expressed in the TAM clusters of all tumor types, suggesting a limitation of the in vitro polarization model in the TME. Notably, SPP1 can be considered as a marker gene of macrophages with angiogenesis function across eight tumor types, including BRCA, pancreatic adenocarcinoma, lung cancer, CRC, uterine corpus endometrial carcinoma, NPC, OV, and THCA.

### Immune microenvironment, dynamic phenotypic changes, and response to immunotherapy by single-cell sequencing

Immune checkpoint blockade (ICB) immunotherapy has introduced a new era of antitumor treatment. It is known that responsiveness to ICB treatment is determined by the preexisting TME and peripheral immuno-compartment of patients. Single-cell analysis is being exploited to investigate whether certain cell populations with multi-parameter defined identities are related to responsiveness or resistance to ICB treatment. Applying single-cell sequencing to predictive biomarker analysis has revealed several translational and clinical insights into ICB-induced tumor control across a range of tumor types. These include melanoma,^23,206^ NSCLC,^207^ glioblastoma,^208^ renal cell carcinoma, squamous cell carcinoma,^209^ bladder cancer,^207^ prostate cancer,^210^ breast cancer,^211^ and urothelial cancer.^212^

Several studies in pre-clinical mouse models have indicated the critical role of the CXCR5/TCF1^+^ subset of CD8^+^ T cells in sustaining a prolonged response to ICB immunotherapy.^213–217^ Studies have demonstrated stem cell-like properties in these cells, characterized by lower expression levels of inhibitory checkpoint molecules such as PD-1, LAG3, TIM-3, and 2B4, and potent self-renewal capacity in the tumor niche. From a phenotypic perspective, this CD8^+^ T cell subset displayed common properties with tumor-residing GZMK^+^ effector memory CD8^+^ T cells and MKi67^+^ expanded CD8^+^ intratumoral T cells. These support the effector memory origin of these cells.^134,158,181^ These findings are consistent with the fact that stem cell-like or memory-like intratumoral CD8^+^ T cells are crucial for effective tumor immunology^218^ and immunotherapy.^219^ In response to ICB treatment, while maintaining self-renewal, stem cell-like CD8^+^ T cells differentiate to yield enough cytotoxic cells with negative TCF1 and high inhibitory checkpoint expression. The CXCR5/TCF1^+^CD8^+^ T cell subset is indispensable for successful ICB immunotherapy. In an ICB-treated B16-bearing mouse model, diphtheria toxin-mediated ablation of TCF1^+^CD8^+^ T cells could not completely abolish tumor control. This suggests that TCF1^+^CD8^+^ T cells are not the only important subset in ICB treatment.^216^ In addition to TCF1^+^CD8^+^ stem cell-like memory cells, tissue-resident memory T cells (T_rm_) can also be important for supporting T cell reinvigoration during ICB immunotherapy.

CD4 T cells are the hub components of the fine-tuned network of the adaptive immune response. Accumulating evidence indicates that in addition to CD8^+^ T cells with direct cytolytic activity, CD4^+^ T cells play an orchestrated role in modulation of tumor immunology and immunotherapy. Facilitated by single T cell analysis by RNA sequencing and TCR tracking (STARTRAC) analysis, Zhang et al. found that Th1 cells are abundant in MSI and sparse in MSS-CRC patients; this is closely associated with responsive or resistant ICB treatment.^181^ Conversely, Th17 cells, showing a paradoxical function in tumor immunology, were significantly enriched in patients with MSS-, but not in patients with MSI-CRC. The balance between Th1/Th17 cell polarization in bone metastatic TME of castration-resistant prostate cancer is associated with sensitivity to anti-PD-1/anti-CTLA-4 treatment.^220^ Intraosseous intratumoral CD4^+^ T cells tend to polarize to the Th17 lineage rather than to the Th1 lineage in the subcutaneous lesion via an osteoclast-associated TGF-β mechanism. In addition to the classical PD-1/PD-L1/CTLA-4-targeting drug, Th1-like CD4^+^ T cells play a critical role in conferring responsiveness to anti-CD40 immunotherapy in both preclinical models and clinical cohorts.^187^ Single-cell sequencing analysis suggests crosstalk between these BHLHE40^+^ Th1-like cells, high in IFN-γ- and CXCL13-producing cells, with cDC1 to synergize with anti-PD-1 administration.

In addition to several helper T cell populations, the presence of MHC-II-restricted cytotoxic CD4^+^ T cells has been documented in various solid tumor types.^134,158,181,193,221–223^ In the case of tumor immunotherapy for patients with bladder cancer, recent single-cell analysis demonstrated a critical role of cytotoxic CD4^+^ T cells in exerting anti-PD-L1-mediated tumor elimination in an MHC-II-restricted manner.^193^ In tumor-bearing mouse models, CD4^+^ T cells can harbor the properties of both cytotoxic and helper cells at the same time.^224^ These multifaceted CD4^+^ T cells are characterized by high expression of cytolytic mediators (GzmB, IFN-γ, and TNF-α), which are independently controlled by T-bet and Blimp-1.

The classic model of tumor immunology holds that tumor-specific naïve T cells are mainly primed in the tumor-draining lymph node. This leads to expression of activation markers, differentiation to effector phenotypes, and recruitment to the tumor site. In addition to local antitumor immunity in the TME, a systemic immune response was evident following effective ICB therapy.^225^ In an organism-wide genetically engineered model, Spitzer et al. demonstrated that tumor eradication following ICB therapy requires immune activation in the peripheral compartment.^225^ Moreover, emerging evidence further supports that T cells from adjacent or peripheral tissues are essential for effective responses to ICB immunotherapies.^135,226–228^ Single-cell sequencing analysis is being used to investigate the association between the response to ICB therapy, preexisting T-cell reinvigoration, and with recruitment of novel peripheral tumor-specific T cells. Among the contributions from “local reinvigoration” versus “peripheral recruitment,” T cell repertoire tracking strategy indicates a clonal replacement mechanism (“peripheral recruitment”) in response to ICB therapy. This consists of a terminally differentiated phenotype of intratumoral tumor-specific T cells. In terms of the CD4^+^ T cell subset, peripheral CD4^+^ T cells are also implicated in mediating antitumor immunity when treated with anti-CTLA-4 antibodies, either from mouse models or patients with metastatic melanoma.^225^

### Intra-tumoral cell–cell communications

**Fig. 4:**
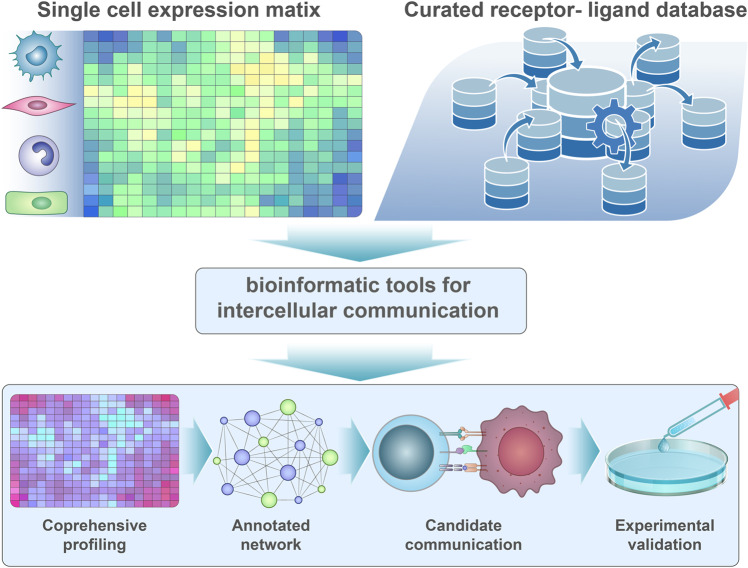
Inferring inter-cellular communication by single-cell sequencing. Inter-cellular contact or transfer of informative material is essential for coordinating the antitumor immune response and the malignant phenotype of tumor cells. Dissecting inter-cellular communication with single-cell sequencing analysis is instructive in understanding active signaling pathways between different cell types, which could eventually be applied to construct a communication network in the tumor immune microenvironment

Many studies have focused on identifying novel cell types or determining the roles of distinct immune cell types in the TME (Fig. 4 and Table 3). However, tumor microenvironments are composed of heterogeneous tumors and immune cells which interact with each other to modulate the cellular network.^229^ In addition to analyzing the characteristics and heterogeneity of the TME, scRNA-seq can be used to infer cell–cell communication between different cell types. Several methods, such as CellPhoneDB,^22^ CellChat,^21^ and NicheNet,^230^ were recently developed for evaluating cellular communication. For inferring inter-cellular communication, the algorithms generally compare the expression level of receptor expression from one cell population and corresponding ligand expression on another cell population with a known gene list of ligand and receptor pairs.^231–234^

**Table 3:** Summary of principle and tools for investigation of intercellular communication by single cell sequencing

Method	Tools	Platform	Characteristic
Differential combinations	CellTalker	R	1. Differential ligand–receptor pairs can be calculated. 2. Capture highly abundant ligand–receptor pairs via comparative analysis.
	iTALK	R	
	PyMINEr	Python	
Expression permutation	CellChat	R and Web	1. Discard random noise results via permutation test. 2. Cluster-to-cluster communication is inferred.
	CellPhoneDB	Python and Web	
	Giotto	R	
	ICELLNET	R	
	SingleCellSignalR	R	
	ProxmID	Software	
	CSOmap	Matlab	
	Cell2Cell	Python	
	MISTY	R	
	stLearn	Python	
	SVCA	R and Python	
Graph or network	CCCExplorer	Software	1. With a prior model, the inference is beyond ligand–receptor interactions and incorporates intracellular signaling. 2. Inference of cell-to-cell communication is possible. 3. Signaling pathway information can also be used.
	NicheNet	R	
	SoptSC	Matlab and R	
	SpaOTsc	Python	
	COMUNET	R	
	NATMI	Python	
Tensor based	scTensor	R	Detect many-to-many CCC involving multiple cell clusters rather than one-to-one CCC.

Based on this database, Ji et al. found that tumor-specific keratinocytes, a subset of keratinocytes in cutaneous squamous cell carcinoma, interact with CAFs, endothelial cells, macrophages, and MDSCs, which influence autocrine and paracrine responses.^235^ Additional ligands from TAMs and MDSCs to tumor-specific keratinocytes contained ITGA3 and ITGB1, indicating their association with EMT and epithelial tumor invasion.

In bladder cancer, inflammatory CAFs (iCAFs) have the largest number of potential ligand–receptor pairs with other cell types, exhibiting particularly strong interactions with endothelial cells.^194^ VEGFA, VEGFB, and FGFR1, produced by iCAFs, bind to endothelial and tumor cells, suggesting that iCAFs promote the proliferation of tumor and stromal cells. Additionally, the receptor CXCL12 is highly expressed in iCAFs and affects immune cell infiltration.

Zhang et al. constructed a computational model by combining scRNA-seq and The Cancer Genome Atlas bulk RNA-seq data to examine cell–cell communication in CRC.^187^ The authors first identified classical cell–cell interactions between fibroblasts and endothelial cells in the tumor and between follicular B cells and Tfh cells in a normal site, indicating that the identification of cell–cell communication using the model was robust and meaningful. In the tumor tissue, TAMs and cDCs became the core of communications, and C1QC^+^ TAMs and two groups of cDCs interacted with T cell subsets, implicating the regulation of antitumor T cell function. Another study of CRC identified several interactions between tumor cells and myofibroblasts, SPP1^+^ macrophages, CD8^+^ T cells, and Treg cells.^182^ Notably, the connection between IgA^+^ plasma cells and CD4^+^ T cells, which supports the mucosal antibody response at the normal site, was weaker than that at the tumor site. Furthermore, the comparison of the CMSs revealed that tumor cells play important roles in the formation of TME.

In gastric tumors, Sathe et al. discovered that the interaction between stromal cells and other cell types exhibited the highest enrichment.^196^ Ligands on stromal cells were connected to EGFR and MET on epithelial cells to promote tumor proliferation. LGR4-RSPO3 is a paired ligand–receptor on epithelial cells and fibroblasts that may regulate stemness.

Zhang et al. investigated cell–cell communication between lymphocytes in HCC, specifically T cells and DC cells.^166^ All DC subsets communicated with Tex cells, proliferative T cells, and Treg cells via the CD28/B7 family and IL-15. LAMP3^+^ interacts with multiple T cells through various ligand–receptor pairs, such as CCL19-CCR7 for CD4^+^ T cells, PD-L1-PD-1 for Tex cells, and effector memory T cells, suggesting that LAMP3^+^ DCs influence T cell function. Additionally, LAMP3^+^ DCs communicate with NK cells through IL-15 and NECTIN2, suggesting that these molecules regulate NK cells.

In head and neck squamous cell carcinoma, Cilo et al. mapped possible cell–cell interactions in human papillomavirus (HPV)^-^ and HPV^+^ HNSCC, respectively.^236^ Almost all immune cell types from HPV^-^ tumors showed unique ligand–receptor pairs versus other cell types, whereas unique communications of HPV^+^ tumors were predicted across pDCs, CD14^+^ cells, CD16^+^ cells, and DCs.

Kim et al. demonstrated that a novel cancer cell type, tS2, strongly interacts with myeloid and stromal cells in the progression or metastases of lung adenocarcinoma.^140^ The most important communication was between tS2 and mo-Macs, whereas the most important communication in the immune cell network was between mo-Macs and CD8^+^ T cells. In primary lung adenocarcinoma, ligand–receptor pairs of growth factors were the most significant between mo-Macs and tS2 cells. Notably, communication between mo-Macs and exhausted CD8^+^ T cells was more complex and included both activating pairs and inhibitory pairs.

Overall, an increasing number of studies have focused on cell–cell communication within the tumor microenvironment or between the tumor and immune cells. These studies have improved understanding of the TME and provided opportunities to develop immunotherapy strategies

### Limitations of single-cell sequencing in cancer research

With continuous advancements in high-throughput omic technology, various bioinformatic tools have recently emerged. At the single-cell level, high-throughput genomics, transcriptomics and epigenomics have been continuously improved. However, these technical methods have limitations that must be overcome.

First, at the transcriptome level, scRNA-seq data are inherently noisy and sparse. Eukaryotic transcription occurs in a pulsed manner rather than at a consistent basal rate.^237,238^ Therefore, when a “0” value is obtained in sequencing results, it may not indicate an inactive state in the cell. In fact, the value of “0” may also imply that the gene is activated but not detected due to burst kinetics, improper sampling time, or technical defects.

Secondly, cells captured using single-cell technology may not represent cells in the body. For example, during the processing of brain tissue into a single-cell suspension, neurons are more likely to be lost than glial cells, leading to deviation between cell composition obtained in the analysis and actual cell composition.^239,240^ In response to this situation, single-nucleus analysis has become a useful alternative. This approach effectively reduces the loss of specific cell types or interference with gene expression but shows lower transcriptome coverage. The specific sample collection and processing procedures also affect the results. scRNA-seq relies on qualified single-cell preparations, which can be obtained from peripheral blood but are typically not easy to isolate from solid tumor mass. In addition, differences in single-cell separation methods and sample storage conditions affect sequencing results.^241^ For example, in blood samples, inconsistencies in the blood draw time can affect cells transcripts and even lead to the changes in the expression of certain key genes (such as specific markers of immune cells).^242^

In addition, 10X scRNA-seq is one of the most popular single-cell techniques. It is a high-throughput method and uses 5′ or 3′ primers to capture RNA. Somatic single nucleotide variations (SNVs) far from transcript ends due to end sequencing are often difficult to capture. When bioinformatics tools (Freebayes, copyKAT etc.) are used to infer SNVs or copy number variations (CNVs) in these data, the results may be slightly flawed.^243,244^ Therefore, methods such as Smart-seq or FLASH-seq that capture the whole transcriptome are better in this regard.^245,246^ Genotyping of Transcriptomes based on the 10x Genomics platform could be an effective solution to this deficiency.^247^ The existence of mature single-cell DNA sequencing technologies (such as Tapestri solution from Mission Bio) must also be mentioned; these may be the best way to accurately obtain genotypes (SNVs, CNVs etc.) at the single-cell level.^248^

The preparation of single-cell suspensions is an essential step in single-cell omics, however cells lose spatial information during this process. Spatial multi-omics (including spatial transcriptomics^249,250^ and spatial epigenomics^251^) allows researchers to understand the locational context of these cells in tissues while acquiring omics data. Unfortunately, spatial multi-omics has not yet reached the level of single-cell resolution.^252^ ST further accelerates the generation of multimodal omics measurements. Multimodal omics implies that multiple modalities up to thousands of single cells can be measured simultaneously in the same experiment.^235,253^ This technology has significant advantages in study design and bioinformatic analysis. The data coverage of single-cell multi-omics in the measurement is similar to that of scRNA-seq but is sparse and noisy. Therefore, the development of biochemical and molecular-based detection tools is the basis for improving the sensitivity, specificity, and robustness of multimodal omics. Currently, high-throughput multimodal omics analysis remains limited to certain types of omic measurements that are mostly based on RNA. However, high-throughput multimodal omics has great potential, and researchers are developing methods for processing massive numbers of cells in a single experiment.

In both single omics or multimodal omics, powerful computing resources and efficient tools are required to analyze large-scale, high-dimensional data. Different single-cell sequencing technologies have varying sequencing coverage and cell capture efficiencies. Single-cell sequencing platforms and species exhibit substantial variation in heterogeneous resolution, sensitivity, and variability. This greatly limits cross-experiment queries or the establishment of a comprehensive database. At present, more powerful statistical and calculation tools are required so that an increasing number of single-cell data can be better integrated and fully utilized. In addition, rapid and accurate integration of cell metabolomics, proteomics, transcriptomics, epigenetics, and genomics is an extremely complex task. Some existing methods include dimension reduction-based approaches,^38^ similarity-based approaches,^254^ and statistical modeling-based approaches,^255^ a considerable number of which target scRNA-seq. Under the integration requirements of low batch scRNA-seq, the ComBat or Harmony methods perform better,^256,257^ while on the atlas-level scRNA-seq data integration task, scANVI, Scanorama, scVI, and scGen perform well. Batch effects from scATAC-seq data can be effectively removed with LIGER and Harmony.^258^ In addition, methods of Single-Cell Multi-omic Integration have been further developed, including LIGER (for gene expression, epigenetic, or spatial data) and WNN (for CITE-seq data).^259,260^

Finally, the cost of single-cell sequencing is expensive. Before applying single-cell sequencing technology, its technical advantages should be considered. The appropriate number of single-cell sequencing platforms should be selected for the subject, as this is important for supporting hypothesis-driven and well-designed research.

## Conclusion

The rapid development of single-cell sequencing technology and analytical tools makes it possible for oncologists to understand the complexity of the tumor immune microenvironment and the resultant antitumor immune response. The application of single-cell sequencing in tumor immunotherapy will substantially enhance the ability of researchers to discover promising targets to overcome immuno-resistance, investigate the signaling pathway and cellular response caused by these drugs and determine the optimal regimen of immuno-combinational therapy in clinical practice. For research on pre-clinical models, single-cell sequencing enables comprehensive characterization of cellular composition and temporal evolution of tumor cells and infiltrated immune and stromal cells. This helps in validating the pathological relevance of disease models and identifying promising targets for drug development. In terms of translation application, the high-dimensional phenotypic information obtained by single-cell sequencing can be used to identify predictive biomarkers in immunotherapy and propose an instructive companion diagnostic strategy for further clinical testing. We anticipate that single-cell sequence analysis will become an indispensable tool in on the fields of tumor immunology and immunotherapy. The massive phenotypic data and biological insights generated from this study will substantially accelerate the progress of antitumor treatment and improve clinical outcomes for patients with cancer.
